# Poly[bis­(1,3-dimethyl­imidazolidin-2-one)(μ_2_-2,5-dioxidoterephthalato)zirconium(IV)]

**DOI:** 10.1107/S1600536813003449

**Published:** 2013-02-16

**Authors:** Matthias Maercz, David Stephen Wragg, Pascal Daniel Croumbie Dietzel, Helmer Fjellvåg

**Affiliations:** a Centre for Materials Science and Nanotechnology, Department of Chemistry, University of Oslo, PO Box 1126, 0315 Oslo, Norway; b Centre for Materials Science and Nanotechnology &, inGAP National Centre of Research-based Innovation, Department of Chemistry, University of Oslo, PO Box 1126, 0315 Oslo, Norway; c Department of Chemistry, University of Bergen, PO Box 7803, 5020 Bergen, Norway

## Abstract

In the title coordination polymer, [Zr(C_8_H_2_O_6_)(C_5_H_10_N_2_O)_2_]_*n*_, the Zr^IV^ atom (site symmetry 2) is coordinated by two *O*,*O*′-bidentate 2,5-dioxidoterephthalate (DHTP^4−^) ligands and two *O*-bonded 1,3-dimethyl-2-imidazolidinone (DMI) ligands (the latter in a *cis* orientation) in a distorted ZrO_6_ octa­hedral geometry. The deprotonated hy­droxy and carb­oxy O atoms of the DHTP^4−^ ligand chelate the Zr^IV^ ion *via* a six-membered ring; the dihedral angle between the carboxyl­ate group and the aromatic ring is 14.46 (11)°. The DHTP^4−^ ligand is completed by crystallographic inversion symmetry and coordinates to two Zr^IV^ atoms, thereby forming polymeric zigzag chains propagating in [001].

## Related literature

For examples of DHTP-containing MOFs, see: Dietzel *et al.* (2005 ▶, 2006 ▶). For examples of zirconium MOFs, see: Chavan *et al.* (2012 ▶).
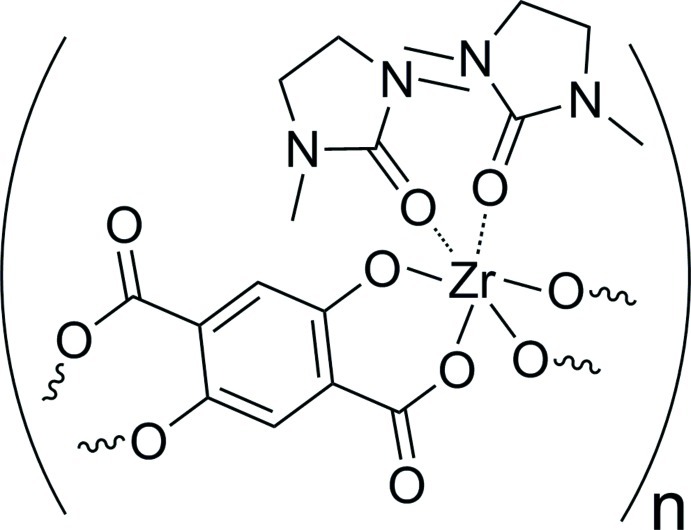


## Experimental

### 

#### Crystal data

[Zr(C_8_H_2_O_6_)(C_5_H_10_N_2_O)_2_]
*M* *_r_* = 513.62
Monoclinic, 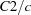

*a* = 17.550 (3) Å
*b* = 8.0828 (12) Å
*c* = 15.425 (2) Å
β = 100.558 (2)°
*V* = 2151.0 (6) Å^3^
*Z* = 4
Mo *K*α radiation
μ = 0.56 mm^−1^
*T* = 293 K
0.10 × 0.10 × 0.08 mm

#### Data collection

Bruker SMART CCD diffractometer
Absorption correction: multi-scan (*SADABS*; Bruker, 2003 ▶) *T*_min_ = 0.946, *T*_max_ = 0.956
5721 measured reflections
2448 independent reflections
1958 reflections with *I* > 2σ(*I*)
*R*_int_ = 0.022

#### Refinement

*R*[*F* ^2^ > 2σ(*F* ^2^)] = 0.038
*wR*(*F* ^2^) = 0.096
*S* = 1.03
2448 reflections
141 parameters
H-atom parameters constrained
Δρ_max_ = 0.36 e Å^−3^
Δρ_min_ = −0.34 e Å^−3^

### 

Data collection: *SMART* (Bruker, 2003 ▶); cell refinement: *SAINT* (Bruker, 2003 ▶); data reduction: *SAINT*; program(s) used to solve structure: *SHELXS97* (Sheldrick, 2008 ▶); program(s) used to refine structure: *SHELXL97* (Sheldrick, 2008 ▶); molecular graphics: *DIAMOND* (Brandenburg, 2006) ▶; software used to prepare material for publication: *publCIF* (Westrip, 2010 ▶).

## Supplementary Material

Crystal structure: contains datablock(s) I, global. DOI: 10.1107/S1600536813003449/hb7025sup1.cif

Structure factors: contains datablock(s) I. DOI: 10.1107/S1600536813003449/hb7025Isup2.hkl

Additional supplementary materials:  crystallographic information; 3D view; checkCIF report

## Figures and Tables

**Table 1:** Selected bond lengths (Å)

Zr1—O1	2.058 (2)
Zr1—O2	2.0215 (17)
Zr1—O3	2.108 (2)

**Fig. 1.**
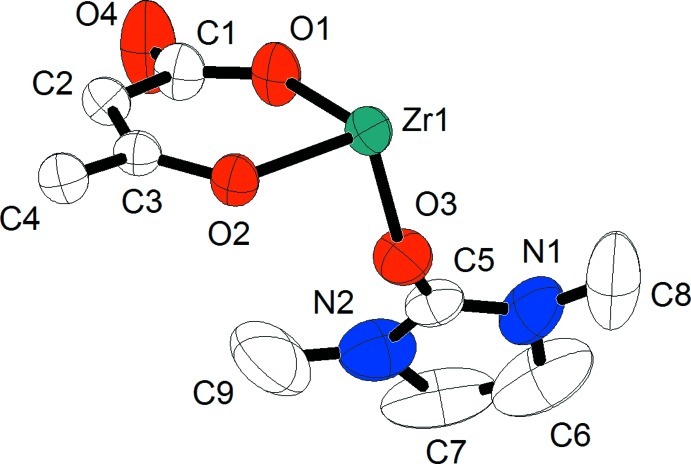
The asymmetric unit of the title compound with 50% probability displacement ellipsoids. Hydrogen atoms are omitted for clarity.

**Fig. 2.**
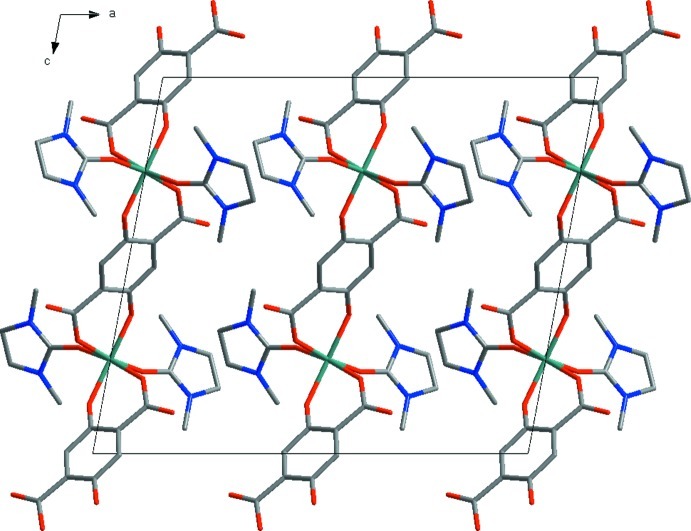
Packing diagram of the title compound viewed along the b axis. Hydrogen atoms are omitted for clarity.

## supplementary crystallographic information

### Comment

The title compound was synthesized as a part of a larger project in which the possibility to form new zirconium containing metal organic frameworks (MOFs) using the linker 2,5-dihydroxyterephthalic acid (DHTP) was investigated. Zirconium containing MOFs (Chavan *et al.*, 2012) using terephthalic acid have shown extraordinary thermal stability whereas DHTP containing MOFs (Dietzel *et al.*, 2005, 2006) have shown remarkable sorption properties.

### Experimental

Zirconium(IV) acetylacetonate (0.098 g, 0.2 mmol) and 2,5-dihydroxyterephthalic acid (0.079 g, 0.4 mmol) were dissolved in in 5 ml 1,3-dimethyl-2-imidazolidinone (DMI) in a Teflon liner of 23 ml volume. The teflon liner was put into a steel autoclave, the steel autoclave was closed and shaken for homogeneity. The mixture was reacted for 3 d at 160°C. Reaction yielded a yellow crystalline substance with larger colorless block shaped crystals. The product was collected by filtration, washed with DMI and dried over night at room temperature in ambient atmosphere.

### Refinement

Hydrogen atoms were placed geometrically in ideal positions and refined using a riding model, the *U*_iso_ set to 1.5 times the thermal parameter of the carbon atom to which they are attached for methyl groups and 1.2 times for other hydrogen atoms.

### Crystal data

**Table:**

[Zr(C_8_H_2_O_6_)(C_5_H_10_N_2_O)_2_]	*F*(000) = 1048
*M**_r_* = 513.62	*D*_x_ = 1.586 Mg m^−^^3^
Monoclinic, *C*2/*c*	Mo *K*α radiation, λ = 0.71073 Å
*a* = 17.550 (3) Å	Cell parameters from 1603 reflections
*b* = 8.0828 (12) Å	θ = 2.4–25.4°
*c* = 15.425 (2) Å	µ = 0.56 mm^−^^1^
β = 100.558 (2)°	*T* = 293 K
*V* = 2151.0 (6) Å^3^	Block, colourless
*Z* = 4	0.10 × 0.10 × 0.08 mm

### Data collection

**Table:**

Bruker SMART CCD diffractometer	2448 independent reflections
Radiation source: sealed tube	1958 reflections with *I* > 2σ(*I*)
Graphite monochromator	*R*_int_ = 0.022
phi and ω scans	θ_max_ = 27.4°, θ_min_ = 2.7°
Absorption correction: multi-scan (*SADABS*; Bruker, 2003)	*h* = −22→22
*T*_min_ = 0.946, *T*_max_ = 0.956	*k* = −10→7
5721 measured reflections	*l* = −19→19

### Refinement

**Table:**

Refinement on *F*^2^	Primary atom site location: structure-invariant direct methods
Least-squares matrix: full	Secondary atom site location: difference Fourier map
*R*[*F*^2^ > 2σ(*F*^2^)] = 0.038	Hydrogen site location: inferred from neighbouring sites
*wR*(*F*^2^) = 0.096	H-atom parameters constrained
*S* = 1.03	*w* = 1/[σ^2^(*F*_o_^2^) + (0.0465*P*)^2^ + 1.4169*P*] where *P* = (*F*_o_^2^ + 2*F*_c_^2^)/3
2448 reflections	(Δ/σ)_max_ < 0.001
141 parameters	Δρ_max_ = 0.36 e Å^−^^3^
0 restraints	Δρ_min_ = −0.34 e Å^−^^3^

### Special details

**Table:**

Geometry. All s.u.'s (except the s.u. in the dihedral angle between two l.s. planes) are estimated using the full covariance matrix. The cell s.u.'s are taken into account individually in the estimation of s.u.'s in distances, angles and torsion angles; correlations between s.u.'s in cell parameters are only used when they are defined by crystal symmetry. An approximate (isotropic) treatment of cell s.u.'s is used for estimating s.u.'s involving l.s. planes.
Refinement. Refinement of *F*^2^ against ALL reflections. The weighted *R*-factor *wR* and goodness of fit *S* are based on *F*^2^, conventional *R*-factors *R* are based on *F*, with *F* set to zero for negative *F*^2^. The threshold expression of *F*^2^ > 2σ(*F*^2^) is used only for calculating *R*-factors(gt) *etc*. and is not relevant to the choice of reflections for refinement. *R*-factors based on *F*^2^ are statistically about twice as large as those based on *F*, and *R*- factors based on ALL data will be even larger.

### Fractional atomic coordinates and isotropic or equivalent isotropic displacement parameters (Å^2^)

**Table:**

	*x*	*y*	*z*	*U*_iso_*/*U*_eq_	
C1	0.10405 (16)	0.0141 (4)	0.37387 (19)	0.0479 (7)	
C2	0.04841 (14)	0.0098 (3)	0.43769 (16)	0.0383 (6)	
C3	−0.01428 (14)	0.1202 (3)	0.43241 (16)	0.0376 (6)	
C4	0.06111 (15)	−0.1071 (3)	0.50427 (17)	0.0411 (6)	
H4	0.1026	−0.1797	0.5068	0.049*	
C5	0.14154 (18)	0.5409 (4)	0.2821 (2)	0.0499 (7)	
C6	0.2275 (3)	0.7161 (6)	0.2379 (5)	0.123 (2)	
H6A	0.2177	0.8342	0.2379	0.148*	
H6B	0.2623	0.6929	0.1973	0.148*	
C7	0.2605 (2)	0.6587 (6)	0.3262 (5)	0.119 (2)	
H7A	0.2711	0.7506	0.3671	0.143*	
H7B	0.3080	0.5972	0.3264	0.143*	
C8	0.0982 (4)	0.6567 (8)	0.1358 (3)	0.145 (2)	
H8A	0.1209	0.7198	0.0943	0.217*	
H8B	0.0560	0.7178	0.1519	0.217*	
H8C	0.0793	0.5535	0.1094	0.217*	
C9	0.2002 (4)	0.4805 (7)	0.4328 (3)	0.138 (2)	
H9A	0.2485	0.5044	0.4710	0.206*	
H9B	0.1938	0.3629	0.4266	0.206*	
H9C	0.1583	0.5257	0.4575	0.206*	
N1	0.15590 (19)	0.6252 (4)	0.2134 (2)	0.0794 (10)	
N2	0.19989 (19)	0.5522 (4)	0.3487 (3)	0.0834 (10)	
O1	0.08341 (11)	0.0970 (3)	0.30113 (13)	0.0528 (5)	
O2	−0.02943 (11)	0.2381 (2)	0.36978 (12)	0.0447 (4)	
O3	0.08044 (12)	0.4612 (3)	0.28274 (14)	0.0596 (6)	
O4	0.16535 (14)	−0.0572 (4)	0.39070 (16)	0.0871 (9)	
Zr1	0.0000	0.26734 (5)	0.2500	0.03931 (15)	

### Atomic displacement parameters (Å^2^)

**Table:**

	*U*^11^	*U*^22^	*U*^33^	*U*^12^	*U*^13^	*U*^23^
C1	0.0396 (16)	0.0571 (18)	0.0517 (16)	0.0091 (13)	0.0210 (13)	0.0038 (13)
C2	0.0318 (14)	0.0452 (15)	0.0397 (13)	0.0033 (11)	0.0117 (11)	−0.0024 (11)
C3	0.0352 (14)	0.0391 (15)	0.0400 (13)	0.0023 (11)	0.0105 (11)	−0.0030 (11)
C4	0.0327 (14)	0.0481 (16)	0.0448 (14)	0.0084 (12)	0.0135 (11)	−0.0006 (12)
C5	0.0468 (17)	0.0397 (16)	0.0657 (19)	0.0011 (13)	0.0169 (15)	−0.0060 (14)
C6	0.085 (4)	0.077 (3)	0.232 (8)	−0.017 (3)	0.090 (5)	−0.004 (4)
C7	0.041 (2)	0.080 (3)	0.232 (7)	−0.007 (2)	0.014 (3)	−0.057 (4)
C8	0.199 (7)	0.143 (5)	0.090 (4)	−0.003 (5)	0.021 (4)	0.057 (4)
C9	0.185 (6)	0.136 (5)	0.071 (3)	0.039 (4)	−0.033 (3)	−0.023 (3)
N1	0.081 (2)	0.065 (2)	0.103 (3)	−0.0082 (17)	0.046 (2)	0.0122 (17)
N2	0.062 (2)	0.074 (2)	0.105 (3)	−0.0014 (17)	−0.0073 (19)	−0.0117 (19)
O1	0.0457 (12)	0.0672 (14)	0.0519 (11)	0.0144 (10)	0.0262 (9)	0.0110 (10)
O2	0.0443 (11)	0.0482 (11)	0.0451 (10)	0.0089 (9)	0.0177 (8)	0.0031 (8)
O3	0.0546 (13)	0.0640 (14)	0.0624 (13)	−0.0195 (11)	0.0167 (10)	−0.0042 (11)
O4	0.0589 (15)	0.133 (2)	0.0792 (17)	0.0487 (16)	0.0389 (13)	0.0420 (16)
Zr1	0.0351 (2)	0.0449 (3)	0.0405 (2)	0.000	0.01366 (15)	0.000

### Geometric parameters (Å, º)

**Table:**

C1—O4	1.206 (3)	C7—H7A	0.9700
C1—O1	1.300 (3)	C7—H7B	0.9700
C1—C2	1.509 (3)	C8—N1	1.442 (6)
C2—C4	1.383 (4)	C8—H8A	0.9600
C2—C3	1.407 (3)	C8—H8B	0.9600
C3—O2	1.348 (3)	C8—H8C	0.9600
C3—C4^i^	1.391 (3)	C9—N2	1.419 (6)
C4—C3^i^	1.391 (3)	C9—H9A	0.9600
C4—H4	0.9300	C9—H9B	0.9600
C5—O3	1.253 (3)	C9—H9C	0.9600
C5—N2	1.314 (4)	Zr1—O1	2.058 (2)
C5—N1	1.322 (4)	Zr1—O2	2.0215 (17)
C6—N1	1.445 (6)	Zr1—O3	2.108 (2)
C6—C7	1.455 (8)	Zr1—O2^ii^	2.0215 (17)
C6—H6A	0.9700	Zr1—O1^ii^	2.058 (2)
C6—H6B	0.9700	Zr1—O3^ii^	2.108 (2)
C7—N2	1.458 (6)		
			
O4—C1—O1	121.9 (2)	H8B—C8—H8C	109.5
O4—C1—C2	120.5 (3)	N2—C9—H9A	109.5
O1—C1—C2	117.6 (2)	N2—C9—H9B	109.5
C4—C2—C3	119.6 (2)	H9A—C9—H9B	109.5
C4—C2—C1	117.7 (2)	N2—C9—H9C	109.5
C3—C2—C1	122.7 (2)	H9A—C9—H9C	109.5
O2—C3—C4^i^	119.5 (2)	H9B—C9—H9C	109.5
O2—C3—C2	122.6 (2)	C5—N1—C8	123.3 (4)
C4^i^—C3—C2	117.9 (2)	C5—N1—C6	109.7 (4)
C2—C4—C3^i^	122.5 (2)	C8—N1—C6	124.9 (5)
C2—C4—H4	118.7	C5—N2—C9	123.9 (4)
C3^i^—C4—H4	118.7	C5—N2—C7	110.6 (4)
O3—C5—N2	125.1 (3)	C9—N2—C7	125.3 (5)
O3—C5—N1	124.1 (3)	C1—O1—Zr1	137.87 (16)
N2—C5—N1	110.8 (3)	C3—O2—Zr1	133.46 (15)
N1—C6—C7	105.1 (4)	C5—O3—Zr1	156.5 (2)
N1—C6—H6A	110.7	O2—Zr1—O2^ii^	166.56 (10)
C7—C6—H6A	110.7	O2—Zr1—O1^ii^	89.38 (7)
N1—C6—H6B	110.7	O2^ii^—Zr1—O1^ii^	81.62 (7)
C7—C6—H6B	110.7	O2—Zr1—O1	81.62 (7)
H6A—C6—H6B	108.8	O2^ii^—Zr1—O1	89.38 (7)
C6—C7—N2	103.2 (4)	O1^ii^—Zr1—O1	96.05 (12)
C6—C7—H7A	111.1	O2—Zr1—O3	98.06 (8)
N2—C7—H7A	111.1	O2^ii^—Zr1—O3	91.94 (8)
C6—C7—H7B	111.1	O1^ii^—Zr1—O3	170.80 (8)
N2—C7—H7B	111.1	O1—Zr1—O3	90.42 (9)
H7A—C7—H7B	109.1	O2—Zr1—O3^ii^	91.94 (8)
N1—C8—H8A	109.5	O2^ii^—Zr1—O3^ii^	98.06 (8)
N1—C8—H8B	109.5	O1^ii^—Zr1—O3^ii^	90.42 (9)
H8A—C8—H8B	109.5	O1—Zr1—O3^ii^	170.80 (8)
N1—C8—H8C	109.5	O3—Zr1—O3^ii^	83.95 (13)
H8A—C8—H8C	109.5		
			
O4—C1—C2—C4	−14.8 (4)	C6—C7—N2—C9	−172.3 (5)
O1—C1—C2—C4	165.3 (3)	O4—C1—O1—Zr1	−166.5 (3)
O4—C1—C2—C3	164.4 (3)	C2—C1—O1—Zr1	13.3 (5)
O1—C1—C2—C3	−15.4 (4)	C4^i^—C3—O2—Zr1	−160.28 (19)
C4—C2—C3—O2	179.3 (2)	C2—C3—O2—Zr1	20.8 (4)
C1—C2—C3—O2	0.1 (4)	N2—C5—O3—Zr1	−108.5 (6)
C4—C2—C3—C4^i^	0.4 (4)	N1—C5—O3—Zr1	71.3 (7)
C1—C2—C3—C4^i^	−178.8 (3)	C3—O2—Zr1—O2^ii^	29.7 (2)
C3—C2—C4—C3^i^	−0.4 (5)	C3—O2—Zr1—O1^ii^	77.5 (2)
C1—C2—C4—C3^i^	178.9 (3)	C3—O2—Zr1—O1	−18.7 (2)
N1—C6—C7—N2	−6.1 (5)	C3—O2—Zr1—O3	−108.0 (2)
O3—C5—N1—C8	10.0 (6)	C3—O2—Zr1—O3^ii^	167.9 (2)
N2—C5—N1—C8	−170.2 (4)	C1—O1—Zr1—O2	0.7 (3)
O3—C5—N1—C6	174.0 (3)	C1—O1—Zr1—O2^ii^	−169.3 (3)
N2—C5—N1—C6	−6.1 (4)	C1—O1—Zr1—O1^ii^	−87.7 (3)
C7—C6—N1—C5	7.7 (5)	C1—O1—Zr1—O3	98.8 (3)
C7—C6—N1—C8	171.5 (5)	C1—O1—Zr1—O3^ii^	46.7 (6)
O3—C5—N2—C9	−2.9 (6)	C5—O3—Zr1—O2	139.9 (6)
N1—C5—N2—C9	177.3 (4)	C5—O3—Zr1—O2^ii^	−31.1 (6)
O3—C5—N2—C7	−178.2 (3)	C5—O3—Zr1—O1^ii^	−76.5 (8)
N1—C5—N2—C7	1.9 (4)	C5—O3—Zr1—O1	58.3 (6)
C6—C7—N2—C5	2.9 (5)	C5—O3—Zr1—O3^ii^	−129.0 (6)

Symmetry codes: (i) −*x*, −*y*, −*z*+1; (ii) −*x*, *y*, −*z*+1/2.
